# Clinical and laboratory characteristics of severe dengue and its comorbidities in Dhaka: a case–control study

**DOI:** 10.3389/fpubh.2026.1944846

**Published:** 2026-10-14

**Authors:** Mohammad Nayeem Hasan, Mahfujur Rahman Himel, Shakera Begum, Farjana Jahan, Sultana Akter, Raisul Islam, Kanis Fatama Ferdushi, Iffat Jahan Ivy, Mahmuda Afrin, Jubayer Mumin, Md. Aminul Islam

**Affiliations:** 1 Department of Statistics, Shahjalal University of Science and Technology, Sylhet, Bangladesh; 2 Department of Social Work, Shahjalal University of Science and Technology, Sylhet, Bangladesh; 3 Department of Statistics, Western Michigan University, Kalamazoo, MI, United States; 4 Department of Biostatistics, Augusta University, Augusta, GA, United States; 5 College of Medicine and Life Sciences, University of Toledo, Toledo, OH, United States; 6 Department of Public Health, North South University, Dhaka, Bangladesh; 7 Department of Aquaculture, Bangladesh Agricultural University, Mymensingh, Bangladesh; 8 Department of Physiology and Pharmacology, Karolinska Institutet, Stockholm, Sweden; 9 Department of Global Public Health, Karolinska Institutet, Stockholm, Sweden; 10 Research Division, Platform Doctors Foundation, Dhaka, Bangladesh; 11 Advanced Molecular Lab, Department of Microbiology, President Abdul Hamid Medical College, Kishoreganj, Bangladesh; 12 COVID-19 Diagnostic lab, Department of Microbiology, Noakhali Science and Technology University, Noakhali, Bangladesh

**Keywords:** Bangladesh, case–control, clinical characteristics, dengue, retrospective study, severe dengue

## Abstract

**Background:**

The prevalence of dengue in Bangladesh has markedly risen, resulting in the most lethal epidemic in 2023 and persistently elevated levels in 2024. Regardless of stringent control measures, dengue persists with comorbidities in Dhaka City. This research explored the correlation between laboratory findings and clinical parameters with severe dengue infection.

**Methods:**

A case–control study was performed on 435 dengue patients (145 cases with severe dengue and 290 controls with non-severe dengue) hospitalized in medical college hospitals in Dhaka City from July to December 2024. Participants were matched by age (±5 years), sex, and admission date (±2 weeks). Univariate and multivariable logistic regression analyses were conducted to find out factors associated with dengue severity.

**Results:**

Comorbidities, such as chronic pulmonary disease (AOR: 2.14; 95% confidence interval (CI): 0.90–5.10), were associated with an increased odds of severe dengue. Symptoms such as abdominal pain (AOR: 2.30; 95% CI: 1.45–3.68), lethargy (AOR: 2.70; 95% CI: 1.35–5.47), and nausea (AOR: 2.21; 95% CI: 1.15–4.22) were associated with increased dengue infection severity. Severe dengue cases exhibited an elevation in serum creatinine, ALT, AST, and pulse rate, but total bilirubin, albumin, and globulin levels were associated with a reduced odds of severity.

**Conclusion:**

This study delineates characteristics associated with severe dengue infection, including comorbidities, such as chronic pulmonary disease, and signs and symptoms, such as abdominal pain, lethargy, and nausea. Laboratory results indicating higher serum creatinine, liver enzymes, and pulse rate, coupled with decreased total bilirubin, albumin, and globulin, were significantly associated with severity. These findings emphasize the significance of early surveillance, especially in patients with comorbidities, and offer critical insights for enhancing the management of severe dengue. Future studies should investigate the transition from non-severe to severe dengue.

## 1 Background

Patients diagnosed with dengue are commonly infected through mosquito bites carrying the dengue virus named DENV (1). Dengue fever is widespread in urban and semi-urban areas in tropical and subtropical countries, endangering more than 50% of the global population (2). This development underscored the disease’s increasing prevalence, especially in tropical and subtropical regions where Aedes mosquito vectors flourish (3, 4). The World Health Organization’s (WHO) global dengue monitoring reported 14,284,310 cases and 10,554 deaths worldwide from January 2024 to December 2024 (5). Dengue fever predominantly occurs in 128 countries, encompassing Southeast Asia, the Eastern Mediterranean, and the Western Pacific (2). In 2023, the two principal hotspots for dengue virus circulation are South America and South and Southeast Asia (6). Like other tropical and subtropical nations, dengue fever poses a significant public health challenge in Bangladesh.

During its time as East Pakistan, Bangladesh encountered its first dengue outbreak in 1964, corresponding with the emergence of the phrase “Dacca fever In recent years, Bangladesh has been profoundly afflicted by dengue viruses, ranking among the most affected nations (7). Government surveillance systems have recorded mortality associated with major epidemics. In 2019, Bangladesh experienced a significant dengue epidemic, reporting 101,354 cases and 164 fatalities associated with the disease. As of 2024, the Ministry of Health & Family Welfare of Bangladesh officially reported 101,214 laboratory-confirmed dengue infections and 575 related fatalities (8). Dengue cases and related deaths in Bangladesh increased by 8.3 and 2.2 times, respectively, between the periods 2000–2010 and 2011–2022 (9). This tendency disproportionately affected males and primarily impacted younger age groups (10). Although Dhaka has historically been the most impacted division, it has reported the highest number of cases outside of Dhaka, accounting for 64.82% (*n* = 193,216) of the total cases. Dhaka continues to have a significant number of dengue-related fatalities, with 980 reported deaths and a case fatality rate of 0.89%, in contrast to 0.34% in areas outside Dhaka (11).

Previous reports indicated infections displaying clinical characteristics aligned with dengue fever (12). Most individuals with dengue remain asymptomatic; however, prevalent indicators encompass elevated fever, cephalalgia, myalgia, nausea, and exanthema. The majority recover within 1–2 weeks; however, some may progress to severe dengue and necessitate hospitalization. Recognizing individuals progressing from mild to severe disease poses challenges; yet it is a critical issue since appropriate intervention might avert the advancement of increasingly severe clinical problems (13). Consistent diagnoses of dengue illnesses and campaigns can be regarded as effective measures in significantly reducing fatality rates (14). However, it appears that the authorities are insufficiently addressing the issue (15, 16). This was evident in the absence of comprehensive public health awareness programs. It is essential to promptly commence campaigns, concentrating on early detection and the delivery of supportive therapeutic treatment. Facilitating timely access to suitable care for dengue patients along with primary healthcare providers can reduce mortality rates (17).

However, there is a scarcity of literature examining the interaction between demographic characteristics, clinical features, comorbidities, and the prognosis/complications of dengue in Bangladesh during recent epidemics. The topic appears to lack focus following the 2019 outbreaks; yet, in recent years, dengue has exceeded all cumulative records since 2000, necessitating further research to draw the attention of policymakers. Understanding this link is crucial for identifying high-risk dengue patients. The multiple warning indicators are essential for quickly identifying severe cases, ensuring timely treatment, preventing unnecessary hospitalizations, and reducing the disease’s case fatality rate. The major objective of this study is to examine the correlation between several clinical and laboratory features, comorbidities, and severe instances of dengue among 2024 dengue patients.

## 2 Materials and methods

This study complied with the STROBE guidelines for reporting case–control studies in epidemiology (Supplementary Table S1).

### 2.1 Study settings

This study was conducted based on four government hospitals in Dhaka, Bangladesh, during the peak months of dengue outbreaks from July 2024 to December 2024. The healthcare support provided to dengue patients in Dhaka comes from Dhaka Medical College Hospital, Shaheed Suhrawardy Medical College and Hospital, Mugda Medical College Hospital, and Mohakhali DNCC Hospital. Dhaka city was chosen as the study area; it accounts for about 40% of the nation’s total dengue cases, according to the 2024 database (8).

### 2.2 Sampling design and sample size

The study included 435 patients to examine potential associated factors for severe dengue fever. All cases were NS1 antigen test–confirmed dengue patients who were hospitalized at the participating hospital(s) until sample collection was completed. Patients were classified as 145 severe cases and 290 non-severe controls. Controls were selected using frequency matching on age (±5 years), sex, and hospital admission date (±2 weeks) to address potential outcome differences across age groups and between sexes. The study recruited patients aged 18 years and older with severe and non-severe dengue defined using the WHO 2009 Dengue Classification (13). Severity was determined using explicit WHO 2009 criteria and required documented clinical evidence of at least one of the following: (1) severe plasma leakage causing shock or respiratory distress; (2) severe bleeding requiring clinical intervention (for example, blood transfusion or invasive hemostatic procedures); or (3) severe organ impairment (hepatic, renal, neurological, or cardiac), as documented by treating physicians in medical records. Classification reflected overall clinical judgment and disease progression, not isolated laboratory values alone. Severity classification was based on documented clinical criteria and physician assessment according to the WHO 2009 classification, rather than on laboratory abnormalities alone.

At participating hospitals, confirmed dengue patients were admitted if they had WHO-defined warning signs, significant comorbidities, concerning laboratory abnormalities, or physician/family concern requiring closer monitoring. Some hospitalized patients without warning signs were also included and were classified as “dengue without warning signs” if they did not progress to severe disease. Not all severe dengue patients required ICU admission. ICU placement depended on clinical judgment, hemodynamic instability, need for organ support, or other life-threatening complications. Patients with severe manifestations who remained hemodynamically stable could be managed in high-dependency or general wards. Therefore, ICU admission was not used to define severity; severity classification followed the World Health Organization 2009 dengue criteria to minimize misclassification bias.

The required sample size was calculated using the EPITOOLS online calculator for case–control studies (18). Assuming 80% power, a 95% confidence level, a minimum expected odds ratio of 2, a 1:2 case–control ratio, and an exposed control proportion of 27%, the required sample size was 432 participants (144 cases and 288 controls). The 1:2 ratio was chosen to increase power while maintaining feasibility because severe dengue was less frequent than non-severe dengue, and this ratio is commonly used in hospital-based case–control studies. The exposed control proportion (27%) reflected the prevalence of hypercholesterolemia among adults in Bangladesh, the highest prevalence among the comorbidities assessed (19).

### 2.3 Data collection

Data was collected by trained research assistants who received structured training on the study protocol, clinical definitions, and standardized data extraction procedures. Electronic medical records from the hospital were utilized to collect administrative, laboratory, and microbiological data. Hematological, biochemical, and vital sign measurements were recorded at the time of hospital admission, which typically coincided with the critical phase of illness. Peak laboratory values during hospitalization were not systematically available and therefore were not used in the analysis. Furthermore, 10% of the cases were randomly chosen for duplicate data entry by a different research assistant, and any inconsistencies were resolved through an independent examination of medical case notes by one of the authors. The extracted data was anonymized for analysis. A validated and standardized questionnaire was used, and clinical records were reviewed by physicians with experience in dengue case management to ensure accuracy.

#### 2.3.1 Outcome variables

The primary outcome variable was dengue disease severity, categorized as severe dengue (cases) and non-severe dengue (controls).

#### 2.3.2 Explanatory variables

A variety of potential factors associated with severe dengue have been examined, including demographic variables such as marital status, educational attainment, and monthly income. Age, sex, and hospital admission date were not considered as explanatory variables due to frequency matching. Furthermore, pre-existing medical problems such as obesity, diabetes, hypertension, chronic lung disease, and ischemic heart disease were examined. The study also examined the signs and symptoms exhibited upon admission with clinical warning signs listed in the WHO 2009 dengue guidelines (e.g., abdominal pain, vomiting, lethargy, and hemorrhage), and diarrhea, headache, rash, chills and rigors, nausea, and musculoskeletal pain, initially evaluated as associated factors based on admission data. Explanatory variables also included hematological parameters measured at admission, namely white blood cell count (WBC), platelet count (thrombocytes), and hemoglobin (Hb). Biochemical variables comprised serum creatinine, total bilirubin, alanine aminotransferase (ALT), aspartate aminotransferase (AST), albumin, and globulin. In addition, pulse rate at admission was included as a physiological variable. All measurements were recorded at the time of hospital admission using a standardized case report form (CRF) to ensure consistent data collection.

#### 2.3.3 Statistical analysis

This study employed chi-square tests for categorical variables and the Wilcoxon rank-sum test for continuous variables. Comparative analyses were performed for unmatched variables only. Age, sex, and hospital admission date were excluded from statistical comparison due to prior frequency matching. Univariate logistic regression models were employed to investigate the association between severe dengue and its associated factors. An arbitrary *p*-value threshold (<0.20) was employed as a criterion for the inclusion of covariates in the multivariate models (20). Sensitivity analyses were also conducted by sequentially excluding laboratory variables (AST, ALT, creatinine) and WHO warning signs (e.g., abdominal pain, vomiting, lethargy, hemorrhage) from the multivariable regression model to assess the robustness of associations and minimize potential incorporation bias or circularity.

In univariate analysis, each variable is separately included in the logistic regression model, yielding a crude odds ratio (COR). The multivariable logistic regression model was employed to provide an adjusted odds ratio (AOR), considering numerous variables concurrently. All tests were conducted at a 5% significance level and presented with COR, AOR, 95% confidence interval (CI), and *p*-value. There were no missing observations for the variables included in the statistical analyses; therefore, no imputation was required. We utilized the statistical tools R, version 3.6.0, and RStudio, version 3.5.2.2 for the analyses (21).

#### 2.3.4 Model evaluation

To evaluate multicollinearity in the final model, a threshold of 4.00 was used for the variance inflation factor (VIF), adhering to the methods specified by Kim (22). All variables were incorporated in this phase of the model, as the VIF value for each variable was below 4.00. To assess the discrimination and calibration of the multivariable logistic regression model, the area under the receiver operating characteristic curve (AUROC) and the Hosmer–Lemeshow goodness-of-fit test were evaluated. Improved model performance is shown by elevated AUROC values. A diminished *P*-value within the ROC curve indicates the model’s proficient differentiation between two categories, with an area under the curve beyond 0.50 signifying enhanced discrimination (23). The Hosmer-Lemeshow goodness-of-fit test assesses the concordance between model-derived probabilities and actual outcomes. A Hosmer-Lemeshow test, generally evaluated via a goodness-of-fit assessment, produces a (*p >* 0.05) signifying the model’s proficiency in accurately classifying data into outcome categories.

### 2.4 Ethical considerations

The Declaration of Helsinki (1964) and its later revisions’ criteria for research with human subjects were followed in the conduct of this study. Prior to data collection, all patients were informed about the study’s goals, and their verbal agreement was obtained. As part of a continuing partnership, the Institute of Epidemiology, Disease Control and Research (IEDCR) obtained the data from a particular hospital in Dhaka city. Every procedure that involved human subjects complied with the IEDCR study committee’s and Shahjalal University of Science and Technology, Sylhet’s ethical guidelines. The IRB approval reference no. is PS/2024/1/41.

## 3 Results

This study included 145 patients and 290 controls, revealing that severe dengue cases had unique socio-demographic, clinico-laboratory, and comorbidity patterns. Regarding marital status, 59.44% of severe patients were married (*p*-value < 0.001). Chronic pulmonary disease was significantly more common in severe cases (15.86%), with a *p*-value of 0.011. Severe dengue cases were more likely to exhibit abdominal pain (58.3%; *p* < 0.001). Lethargy was observed in 19.44% of severe cases, while the remainder did not exhibit this symptom (*p* < 0.001). Nausea occurred in 18.62% of severe cases, with the majority of severe cases not exhibiting nausea (*p* = 0.011) (Table 1).

**Table 1:** Sociodemographic prevalence, comorbidities, and signs and symptoms of severe and non-severe dengue cases*.

Exposure variables	Severe *n* (%)	Non-severe *n* (%)	Totals *n* (%)	*p*-value
Socio-demographic				
Marital status				
Married	85 (59.44)	120 (41.81)	205 (47.67)	<0.001
Unmarried	58 (40.56)	167 (58 0.19)	225 (52.33)	
Education level				
Postgraduate	7 (4.96)	7 (2.46)	14 (3.29)	0.316
College/University	27 (19.15)	50 (17.61)	77 (18.12)	
High school or less	58 (41.13)	107 (37.68)	165 (38.82)	
No education	49 (34.75)	120 (42.25)	169 (39.76)	
Monthly income				
<15,000 Taka	78 (56.52)	140 (48.95)	218 (51.42)	0.143
15,001–30,000 Taka	27 (19.57)	81 (28.32)	108 (25.47)	
30,000 + Taka	33 (23.91)	65 (22.73)	98 (23.11)	
Comorbidities				
Obesity				
Yes	55 (37.93)	107 (36.90)	162 (37.24)	0.833
No	90 (62.07)	183 (63.10)	273 (62.76)	
Diabetes				
Yes	11 (7.59)	14 (4.83)	25 (5.75)	0.244
No	134 (92.41)	276 (95.17)	410 (94.25)	
Hypertension				
Yes	19 (13.10)	28 (9.66)	47 (10.80)	0.275
No	126 (86.90)	262 (90.34)	388 (89.20)	
Chronic pulmonary disease				
Yes	23 (15.86)	23 (7.93)	46 (10.57)	0.011
No	122 (84.14)	267 (92.07)	389 (89.43)	
Ischemic heart disease				
Yes	14 (9.66)	18 (6.21)	32 (7.36)	0.194
No	131 (90.34)	272 (93.79)	403 (92.64)	
Signs and symptoms				
Abdominal pain				
Yes	84 (58.3)	107 (36.9)	191 (44.01)	<0.001
No	60 (41.7)	183 (63.1)	243 (55.99)	
Diarrhea				
Yes	33 (22.8)	61 (21.03)	94 (21.61)	0.680
No	112 (77.2)	229 (78.97)	341 (78.39)	
Vomiting				
Yes	14 (9.66)	22 (7.58)	36 (8.28)	0.460
No	131 (90.34)	268 (92.41)	399 (91.72)	
Lethargy				
Yes	28 (19.44)	21 (7.27)	49 (11.32)	<0.001
No	116 (80.56)	268 (92.73)	384 (88.68)	
Headache				
Yes	110 (76.39)	225 (77.59)	335 (77.19)	0.780
No	34 (23.61)	65 (22.41)	99 (22.81)	
Rash				
Yes	103 (71.53)	203 (70.00)	306 (70.51)	0.742
No	41 (28.47)	87 (30.00)	128 (29.49)	
Chills and/or Rigors				
Yes	39 (26.90)	79 (27.24)	118 (27.13)	0.939
No	106 (73.10)	211 (72.75)	317 (72.87)	
Nausea				
Yes	27 (18.62)	29 (10.00)	56 (12.87)	0.011
No	118 (81.38)	261 (90.00)	379 (87.13)	
Hemorrhage				
Yes	7 (4.90)	13 (4.50)	20 (4.63)	0.854
No	136 (95.10)	276 (95.50)	412 (95.37)	
Musculoskeletal pain				
Yes	108 (75.0)	207 (71.38)	315 (72.58)	0.426
No	36 (25.0)	83 (28.62)	119 (27.42)	
Total	145 (33.33)	290 (66.67)	435 (100.00)	

*Signs and symptoms were recorded at hospital admission and are presented descriptively. Not all listed symptoms were used as defining criteria for WHO 2009 severity classification.

Table 2 delineates the laboratory findings between severe dengue cases and non-severe controls, highlighting considerable disparities in various clinical parameters. The median white blood cell (WBC) count was markedly reduced in severe cases (4.90) relative to non-severe cases (5.51). Moreover, severe individuals exhibited markedly elevated median serum creatinine levels (132.11) in contrast to non-severe cases (104.80). Total bilirubin levels were elevated in the severe group, with a median of 10.34, in contrast to 12.46 in the non-severe group. The liver enzyme indicators, ALT and AST, were markedly elevated in severe instances. The median AST was 229.39 in the severe group, in contrast to 100.31 in the non-severe group, and the median ALT was 46.66 in severe instances compared to 37.66 in non-severe cases. Severe cases demonstrated markedly reduced median albumin levels (39.78) relative to non-severe cases (42.36), and globulin levels were similarly diminished in severe cases (24.72) compared to non-severe cases (27.53). The median pulse rate was marginally elevated in severe cases (89.68) compared to the non-severe group (88.14).

**Table 2:** Comparison of clinical and laboratory results between severe and non-severe dengue cases*.

Variables	Cases Median (IQR)	Controls Median (IQR)	Totals Median (IQR)	*p*-value
WBCs (cells × 10^9^ /L)	4.90 (3.39–5.76)	5.51 (3.62–7.12)	5.20 (3.52–6.64)	<0.001
Thrombocytes (cells × 10^9^ /L)	85.54 (76.14–93.29)	94.49 (86.16–101.87)	91.55 (84.14–98.34)	<0.001
Hemoglobin (g/dL)	15.36 (12.85–18.12)	15.19 (12.72–17.49)	15.23 (15.06–15.39)	0.264
Serum Creatinine (μmol/L)	132.11 (119.18–148.69)	104.80 (97.78–112.32)	111.01 (105.18–132.96)	<0.001
Total bilirubin (μmol/L)	10.34 (9.37–11.60)	12.46 (11.33–3.69)	11.80 (10.54–13.04)	<0.001
ALT (IU/L)	46.66 (38.54–53.04)	37.66 (32.22–49.82)	40.70 (40.65–45.88)	<0.001
AST (IU/L)	229.39 (210.31–259.54)	100.31 (79.14–123.42)	119.72 (88.19–217.66)	<0.001
Albumin (g/L)	39.78 (37.71–41.85)	42.36 (41.07–43.79)	41.72 (40.35–43.42)	<0.001
Globulin (g/L)	24.72 (22.10–27.06)	27.53 (26.13–28.76)	26.94 (25.32–28.36)	<0.001
Pulse rate (BPM)	89.68 (87.44–91.59)	88.14 (85.81–90.37)	88.58 (86.42–90.91)	<0.001

*Median laboratory values are presented for comparison between groups and do not necessarily represent WHO 2009 severity threshold values at the individual patient level.

Figure 1 shows significant positive correlations between total bilirubin and WBC (*r* = 0.133), albumin (*r* = 0.104), thrombocytes (*r* = 0.222), and globulin (*r* = 0.303); thrombocytes with albumin (*r* = 0.247) and globulin (*r* = 0.296); and serum creatinine with AST (*r* = 0.564), ALT (*r* = 0.123), and pulse rate (*r* = 0.156). Additionally, albumin and globulin were positively correlated (*r* = 0.302), and AST was positively associated with pulse rate (*r* = 0.149). Negative correlations were observed between thrombocytes and serum creatinine (*r* = −0.309); serum creatinine and total bilirubin (*r* = −0.403); AST with albumin (*r* = −0.429) and globulin (*r* = −0.249); and pulse rate with thrombocytes (*r* = −0.123), albumin (*r* = −0.146), globulin (*r* = −0.166), and total bilirubin (*r* = −0.190). WBC also negatively correlated with serum creatinine (*r* = −0.140) and AST (*r* = −0.147). Thrombocytes were negatively correlated with AST (*r* = −0.375) and ALT (*r* = −0.117).

**Figure 1:**
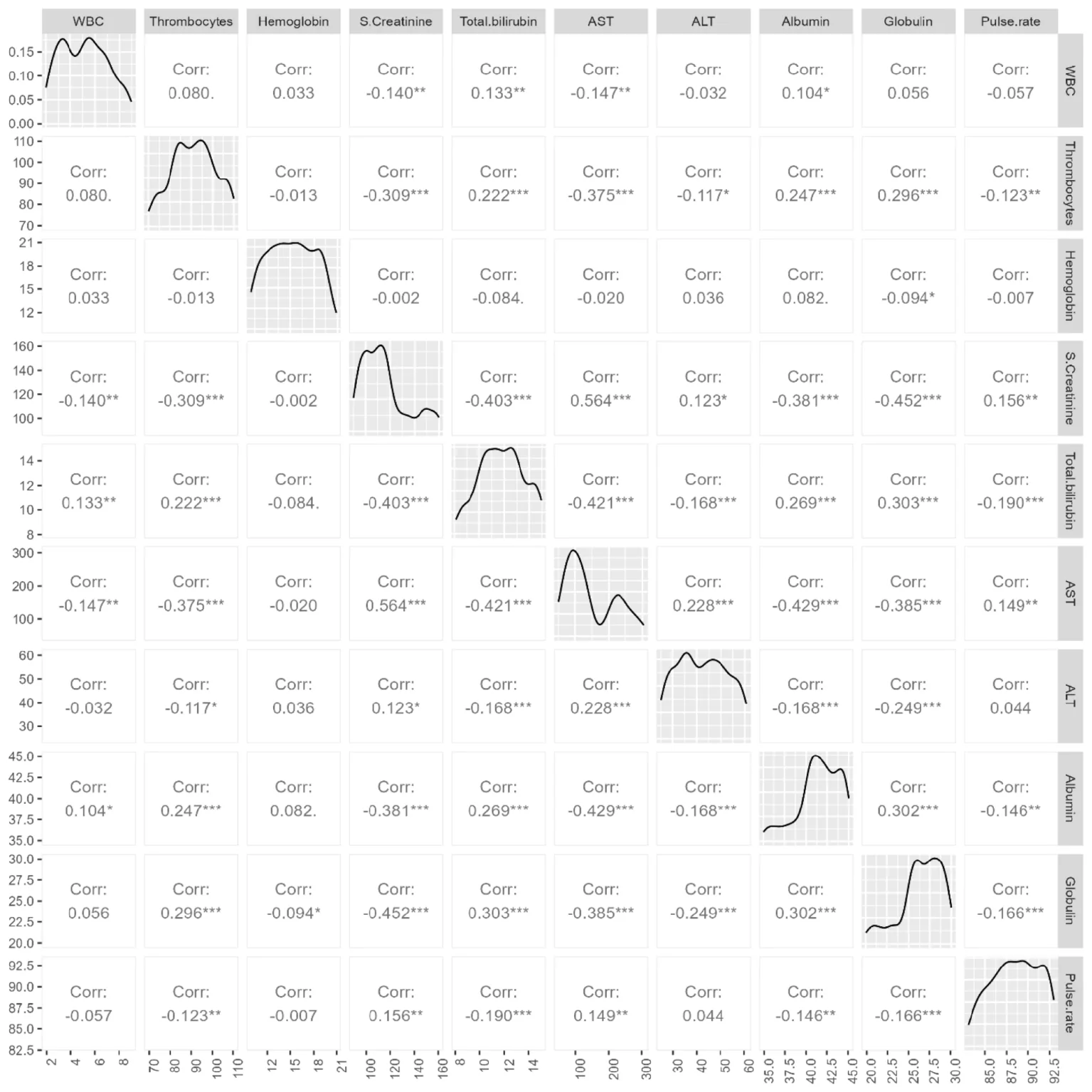
Correlation matrix between various clinical and laboratory parameters of confirmed dengue cases.

Table 3 summarizes the associations between sociodemographic characteristics, comorbidities, signs and symptoms, and dengue severity. In the crude analysis, marital status (married), chronic pulmonary disease, abdominal pain, lethargy, and nausea were significantly associated with higher odds of severe dengue (*p* < 0.05). Other sociodemographic factors, comorbidities such as diabetes, hypertension, ischemic heart disease, obesity, and symptoms including vomiting, diarrhea, headache, rash, chills and rigors, hemorrhage, and musculoskeletal pain were not significantly associated with severity (*p* > 0.05). In both crude and adjusted analyses, married individuals had significantly higher odds of severe dengue compared to unmarried participants (adjusted odds ratio [AOR] 1.79; 95% confidence interval [CI]: 1.11–2.89). Educational level was also associated with severity; participants with a high school education or less were more likely to experience severe dengue than those with no formal education (AOR 1.77; 95% CI: 1.04–3.04). Although chronic pulmonary disease showed a significant crude association with severity, this relationship attenuated after adjustment (AOR 2.14; 95% CI: 0.90–5.10). Among clinical features, abdominal pain (AOR 2.30; 95% CI: 1.45–3.68), lethargy (AOR 2.70; 95% CI: 1.35–5.47), and nausea (AOR 2.21; 95% CI: 1.15–4.22) were associated with increased odds of severe dengue compared with their counterparts. Other examined comorbidities and symptoms did not demonstrate significant associations in the adjusted model.

**Table 3:** Association between the signs and symptoms of severe and non-severe dengue cases, sociodemographics, and comorbidities.

Exposure variables	Univariable model		Multivariable model	
	COR (95% CI)	*P*-value	AOR (95% CI)	*P*-value
Socio-demographic				
Marital status				
Married	2.04 (1.36–3.08)	<0.001	1.79 (1.11–2.89)	0.017
Unmarried	Reference		Reference	
Highest education				
Postgraduate	2.45 (0.80–7.51)	0.110	3.02 (0.92–9.97)	0.066
College/University	1.32 (0.74–2.34)	0.340	1.62 (0.84–3.11)	0.145
High school or less	1.33 (0.84–2.11)	0.228	1.77 (1.04–3.04)	0.036
No education	Reference		Reference	
Monthly income				
<15,000 Taka	1.10 (0.67–1.83)	0.717	1.10 (0.62–1.96)	0.749
15,001–30,000 Taka	0.66 (0.36–1.20)	0.172	0.81 (0.42–1.57)	0.535
30,000 + Taka	Reference		Reference	
Comorbidities				
Obesity				
Yes	1.05 (0.69–1.57)	0.833		
No	Reference			
Diabetes				
Yes	1.62 (0.70–3.65)	0.248		
No	Reference			
Hypertension				
Yes	1.41 (0.75–2.61)	0.276		
No	Reference			
Chronic pulmonary disease				
Yes	2.19 (1.18–4.07)	0.013	2.14 (0.90–5.10)	0.084
No	Reference			
Ischemic heart disease				
Yes	1.61 (0.77–3.34)	0.197	0.84 (0.29–2.31)	0.732
No	Reference			
Signs and symptoms				
Abdominal pain				
Yes	2.39 (1.60–3.61)	<0.001	2.30 (1.45–3.68)	<0.001
No	Reference			
Diarrhea				
Yes	1.11 (0.68–1.78)	0.681		
No	Reference			
Vomiting				
Yes	1.30 (0.63–2.60)	0.461		
No	Reference			
Lethargy				
Yes	3.08 (1.69–5.71)	**<0.001**	2.70 (1.35–5.47)	0.005
No	Reference			
Headache				
Yes	0.93 (0.59–1.51)	0.780		
No	Reference			
Rash				
Yes	1.08 (0.70–1.68)	0.742		
No	Reference			
Chills and rigors				
Yes	0.98 (0.62–1.53)	0.939		
No	Reference			
Nausea				
Yes	2.06 (1.16–3.64)	0.013	2.21 (1.15–4.22)	0.016
No	Reference			
Hemorrhage				
Yes	1.09 (0.40–2.73)	0.853		
No	Reference			
Musculoskeletal pain				
Yes	1.20 (0.77–1.91)	0.426		
No	Reference			

*Clinical warning signs included in this table were evaluated as associated factors based on admission data. Sensitivity analyses excluding WHO warning-sign variables (abdominal pain, vomiting, lethargy, hemorrhage) yielded comparable effect estimates.

In the multivariable analysis of laboratory data, several laboratory parameters were associated with severe dengue. Higher serum creatinine (AOR 1.26; 95% CI: 1.14–1.48; *p* < 0.001), ALT (AOR 1.04; 95% CI: 1.02–1.06; *p* < 0.001), and AST (AOR 1.14; 95% CI: 1.04–1.27; *p* = 0.012) were associated with increased odds of severe dengue. Conversely, higher levels of total bilirubin (AOR 0.44; 95% CI: 0.20–0.79; *p* = 0.015), albumin (AOR 0.64; 95% CI: 0.41–0.91; *p* = 0.026), and globulin (AOR 0.36; 95% CI: 0.17–0.62; *p* = 0.002) were associated with lower odds of severe dengue. Other variables, including WBC, thrombocytes, and pulse rate, were not significant in the adjusted model (Table 4).

**Table 4:** Association between clinical and laboratory results from severe and non-severe dengue cases.

Variables	Univariable model		Multivariable model	
	COR (95% CI)	*p*-value	AOR (95% CI)	*p*-value
WBCs (cells × 10^9^ /L)	0.80 (0.71–0.89)	<0.001	0.76 (0.45–1.19)	0.254
Thrombocytes (cells × 10^9^ /L)	0.89 (0.86–0.91)	<0.001	0.93 (0.84–1.01)	0.115
Hemoglobin (g/dL)	1.04 (0.97–1.12)	0.261		
Serum Creatinine (μmol/L)	1.20 (1.16–1.25)	<0.001	1.26 (1.14–1.48)	<0.001
Total bilirubin (μmol/L)	0.39 (0.32–0.47)	<0.001	0.44 (0.20–0.79)	0.015
ALT (IU/L)	1.05 (1.03–1.07)	<0.001	1.04 (1.02–1.06)	<0.001
AST (IU/L)	1.03 (1.03–1.04)	<0.001	1.14 (1.04–1.27)	0.012
Albumin (g/L)	0.54 (0.47–0.61)	<0.001	0.64 (0.41–0.91)	0.026
Globulin (g/L)	0.53 (0.46–0.60)	<0.001	0.36 (0.17–0.62)	0.002
Pulse rate (BPM)	1.20 (1.11–1.30)	<0.001	1.36 (0.90–2.14)	0.154

Results from sensitivity analyses excluding WHO 2009 warning signs and laboratory variables yielded results consistent with the primary model, indicating that incorporation bias did not materially influence the observed associations. The direction and magnitude of associations remained consistent across models. Effect sizes were attenuated after exclusion of organ-specific markers, but key associations remained statistically significant, suggesting partial but not exclusive dependence on severity-defining variables (Supplementary Tables S2, S3).

The variance inflation factor (VIF) study reveals that all variables in the final models had VIF values below 4.00, indicating no evidence of substantial multicollinearity according to the prespecified threshold. Furthermore, the model demonstrated moderate discrimination, with an AUROC of 71.54%. The Hosmer–Lemeshow goodness-of-fit test did not indicate evidence of poor fit (χ^2^ = 10.46, df = 8, *p* = 0.712) (Table 5 and Figure 2). The model successfully passed the Hosmer and Lemeshow goodness-of-fit test, yielding a value of 10.46 with 8 degrees of freedom (*P* = 0.712), indicating no lack of fitness in the model.

**Table 5:** Assessment of the final model’s predictive accuracy and goodness of fit.

Hosmer and Lemeshow’s goodness of fit test		
X-squared	Degrees of freedom	*P*-value
10.46	8	0.712

Area under the receiver operating characteristic curve (AUROC)	
Value	71.54 (66.16–76.93)%

**Figure 2:**
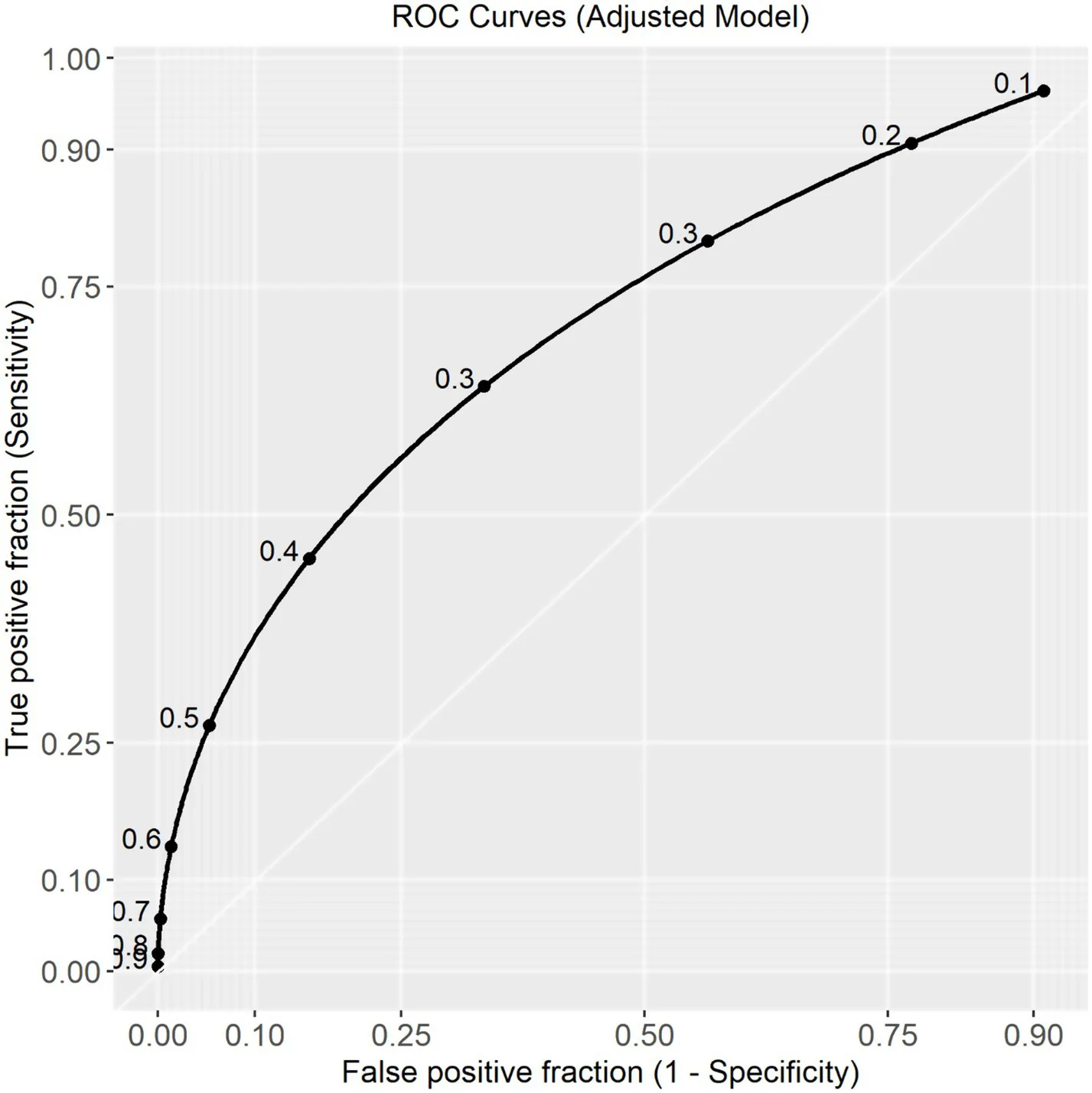
Area under the receiver operating characteristic curve (AUROC) of the final model.

## 4 Discussion

This research assessed the factors associated with severe dengue in 145 cases and 290 controls admitted during the 2024 dengue outbreak in Bangladesh. This study sought to identify associated factors for severe dengue during the outbreak, uncovering a complicated interaction of demographic characteristics, comorbidities, and symptoms. The results highlight the importance of demographic characteristics, comorbidities, and clinical symptoms of severity of dengue, consistent with previous research, while providing new perspectives relevant to the setting of Bangladesh. Our findings indicate that certain sociodemographic factors and clinical features were associated with severe dengue. Specifically, marital status and lower educational attainment, as well as clinical warning signs including abdominal pain, lethargy, and nausea, were associated with increased odds of severe disease. While comorbidities such as chronic pulmonary disease and ischemic heart disease showed significant associations in univariate analyses, they did not retain independent predictive value in the multivariable model. These results highlight the importance of both social and clinical factors in dengue severity and suggest that further research is needed to clarify the mechanisms through which comorbid conditions influence disease progression.

Abdominal pain, lethargy, and nausea were strongly associated with severe dengue in both unadjusted and adjusted models. These symptoms have long been acknowledged as indicators of the development of severe dengue. Some studies identified four significant symptoms associated with an increased odds of severe dengue: nausea, vomiting, bleeding (including gum bleeding, epistaxis, hematemesis, and melaena), abdominal pain, and skin rashes (24–29). Abdominal pain demonstrated a significant correlation with severe outcomes, aligning with prior research indicating it serves as a marker for plasma leakage and approaching shock (30–32). In contrast to our findings, the occurrence of headache was associated with a reduced probability of severe dengue, an intriguing finding supported by recent studies indicating that headache may be inversely associated with disease severity. A retrospective cohort study in Singapore, comprising 82 patients with dengue fever and 1855 individuals with dengue fever, indicated that the presence of headache at presentation was associated with decreased likelihood of developing dengue fever (33). However, a lower likelihood of severe dengue was observed among patients presenting with headaches, although this association was not statistically significant in our study.

Our study demonstrated that the odds of severe dengue were significantly higher in those presenting chronic pulmonary disease and ischemic heart disease. Previous evidence has shown that acute DENV infection is associated with significantly higher odds of new-onset cardiovascular events within 30 days after infection compared with uninfected population-based controls, although the excess burden is relatively modest (34). Diabetes and hypertension have been reported as higher-risk associated factors of adverse outcomes in previous studies; however, although our study did not find a statistically significant association, the magnitude of risk was consistent with previous findings. This corresponds with international research highlighting diabetes as a significant risk factor due to its effects on immunological regulation and endothelial dysfunction, which may aggravate problems related to dengue and prior studies conducted in Bangladesh (35, 36). A study conducted among the Chinese population during the 2007–2008 epidemic, when dengue serotype 2 was predominant, found that hypertension was a risk factor for developing dengue hemorrhagic fever dengue fever (37).

Thrombocytopenia is a common laboratory finding in dengue infection and is often associated with disease severity. In our study, platelet counts were significantly lower among severe dengue cases compared with non-severe cases, although this association did not remain significant after adjustment in the multivariable model. Markers of hepatic injury, including alanine aminotransferase (ALT) and aspartate aminotransferase (AST), were significantly elevated among severe dengue cases, consistent with previous studies reporting liver involvement in severe dengue (38). Both ALT and AST remained independently associated with severe dengue in the adjusted analysis. Additionally, serum creatinine levels were significantly higher in severe dengue cases and remained an independent factor of disease severity. This finding is consistent with previous studies reporting that renal impairment in dengue may result from hypovolemia, shock, rhabdomyolysis, or immune-mediated injury (33). In contrast, lower levels of albumin and globulin were observed among severe cases and remained significantly associated with dengue severity.

Furthermore, a number of viral and host factors, such as the non-structural protein 1 (NS1) antigen, DENV genome variation, subgenomic RNA, antibody-dependent enhancement (ADE), cross-reactive memory T cells, anti-DENV NS1 antibodies, and autoimmunity, are thought to work together to cause severe dengue symptoms in humans (39). A primary infection, which is usually asymptomatic or manifests as a mild feverish illness, is the first time a particular DENV serotype is encountered. But sometimes, especially in babies born to mothers who are DENV-immune, it can result in hemorrhagic fever. There is a greater risk of serious outcomes, such as dengue hemorrhagic fever (DHF) or dengue shock syndrome (DSS), with a secondary dengue infection involving a different serotype (40–42). Following infection, individuals acquire long-term immunity to the infecting serotype but only short-lived cross-protection against other serotypes. Cohort studies suggest that this heterotypic immunity typically wanes within 1–2 years (43). However, the current study could not assess these dynamics due to limited longitudinal follow-up, insufficient serotype-specific diagnostic capacity, and a small cohort with suboptimal sampling intervals. Consequently, more extensive, long-term, serotype-specific studies are essential to better understand the waning of cross-protective immunity and its influence on severe dengue pathogenesis.

### 4.1 Strengths and limitations

This study has several strengths. First, it addresses severe dengue during the 2024 epidemic in Bangladesh using data from 435 hospitalized adults, including 145 severe dengue cases and 290 non-severe dengue controls. Second, frequency matching by age, sex, and hospital admission date improved comparability between severe and non-severe groups. Third, the study incorporated a broad range of sociodemographic characteristics, comorbidities, clinical symptoms, hematological and biochemical parameters, and vital signs, allowing a multidimensional assessment of characteristics associated with severe dengue. Fourth, severe dengue was classified according to the WHO 2009 criteria using documented clinical evidence rather than laboratory abnormalities alone. Finally, standardized data collection, duplicate data entry for a subset of participants, and physician review of medical records were used to improve data quality.

Several limitations should be considered when interpreting the findings. First, the hospital-based case–control design limits generalizability because participants were recruited from selected government hospitals in Dhaka City and may not represent dengue patients managed in outpatient or community settings. This study also was unable to collect patients’ serotypes. Consequently, the findings should be interpreted primarily as associations among hospitalized adults with dengue and may not be directly generalizable to other populations or geographic regions of Bangladesh. Selection bias is also possible because both cases and controls were recruited from hospitalized patients.

Second, given the observational case–control design, the observed associations should not be interpreted as causal relationships. Although laboratory and vital-sign measurements were obtained at hospital admission, severe dengue classification incorporated documented clinical manifestations and disease progression. Therefore, temporality between some laboratory abnormalities and the development of severe dengue cannot be established with certainty, and these measurements should be interpreted as characteristics associated with severe dengue rather than definitive causal predictors of subsequent progression.

Third, residual confounding cannot be excluded. Although demographic, socioeconomic, comorbidity, clinical, and laboratory characteristics were considered in the analyses, information on previous dengue infection or immune status, nutritional status, duration of illness before hospital admission, and treatment received before hospital admission was not comprehensively available. These factors may have influenced disease severity and could therefore contribute to residual confounding. In addition, dengue virus serotypes and primary versus secondary infection could not be determined because diagnosis was based on NS1 antigen positivity. NS1 confirms acute dengue infection but does not permit serotype identification or differentiation between primary and secondary infections, which may influence disease severity.

Fourth, some information and measurement bias cannot be completely excluded because symptoms and comorbidities were obtained from medical records and/or patient-reported information and may have been incompletely documented. Although standardized data collection, duplicate data entry, and physician review were used to improve data quality, retrospective documentation may still contain incomplete or inaccurate information.

Fifth, some comorbidities had relatively small numbers of exposed participants, which may have reduced statistical precision and resulted in wide confidence intervals for their estimated associations. Although variance inflation factor assessment did not indicate substantial multicollinearity in the final models, moderate correlations among some biochemical variables were observed; therefore, some instability in individual coefficient estimates cannot be completely excluded.

Finally, although model discrimination and goodness of fit were assessed using the AUROC and Hosmer–Lemeshow test, the model was not externally validated in an independent population. Therefore, the findings should not be interpreted as a validated clinical prediction tool. Future multicenter prospective studies with larger samples, longitudinal follow-up, serotype-specific testing, assessment of primary versus secondary infection, and external validation are needed to confirm these findings and better evaluate the transition from non-severe to severe dengue.

## 5 Conclusion

This study delineates numerous critical parameters associated with severe dengue, emphasizing essential socio-demographic, clinico-laboratory, and comorbidity patterns. Marital status, monthly income, chronic pulmonary disease, abdominal pain, lethargy, and nausea were all substantially correlated with severe dengue, suggesting that both pre-existing illnesses and particular symptoms are pivotal in ascertaining disease severity. Moreover, test results included platelet count, serum creatinine, total bilirubin, liver enzymes (AST and ALT), albumin, and globulin levels, which were significantly associated with severe dengue. Significantly, increased blood creatinine and liver enzymes, coupled with decreased platelet, albumin, and globulin levels, were more evident in severe cases. These findings underscore the necessity for prompt clinical surveillance, especially for those with comorbidities such as diabetes and hypertension, as well as those exhibiting symptoms including abdominal discomfort, vomiting, lethargy, headache, or rash. These results provide significant insights into the therapeutic management of dengue and establish a foundation for future research on the association of severe disease progression. This research provides new insights into identifying key associated factors for severe dengue, enhancing our comprehension of early detection and treatment. Future research should investigate the transition from non-severe to severe dengue more comprehensively, expanding upon the results of this study.

## Data Availability

The datasets generated and/or analyzed during the current study are available from the corresponding authors upon reasonable request.
