# Supplementary material for: Clinical and laboratory characteristics of severe dengue and its comorbidities in Dhaka: a case–control study
Source: Front Public Health. 2026 Oct 14;14:1944846. doi: 10.3389/fpubh.2026.1944846 (PMC13713829; doi:10.3389/fpubh.2026.1944846)
Supplement: Supplementary file 1 [file Supplementary_file_1.docx]

**Table S1:** STROBE Statement—Checklist of items that should be included in reports of ***case-control studies***

|  | Item No | Recommendation | Page # |
| --- | --- | --- | --- |
| **Title and abstract** | 1 | (*a*) Indicate the study’s design with a commonly used term in the title or the abstract | 1 |
|  |  | (*b*) Provide in the abstract an informative and balanced summary of what was done and what was found | 1 |
| Introduction | | | |
| Background/rationale | 2 | Explain the scientific background and rationale for the investigation being reported | 2 |
| Objectives | 3 | State specific objectives, including any prespecified hypotheses | 2 |
| Methods | | | |
| Study design | 4 | Present key elements of study design early in the paper | 2 |
| Setting | 5 | Describe the setting, locations, and relevant dates, including periods of recruitment, exposure, follow-up, and data collection | 2 |
| Participants | 6 | (*a*) Give the eligibility criteria, and the sources and methods of case ascertainment and control selection. Give the rationale for the choice of cases and controls | 3 |
|  |  | (*b*) For matched studies, give matching criteria and the number of controls per case | 3 |
| Variables | 7 | Clearly define all outcomes, exposures, predictors, potential confounders, and effect modifiers. Give diagnostic criteria, if applicable | 3 |
| Data sources/ measurement | 8* | For each variable of interest, give sources of data and details of methods of assessment (measurement). Describe comparability of assessment methods if there is more than one group | 3 |
| Bias | 9 | Describe any efforts to address potential sources of bias | 3 |
| Study size | 10 | Explain how the study size was arrived at | 2-3 |
| Quantitative variables | 11 | Explain how quantitative variables were handled in the analyses. If applicable, describe which groupings were chosen and why | 3 |
| Statistical methods | 12 | (*a*) Describe all statistical methods, including those used to control for confounding | 3 |
|  |  | (*b*) Describe any methods used to examine subgroups and interactions | N/A |
|  |  | (*c*) Explain how missing data were addressed | 3 |
|  |  | (*d*) If applicable, explain how matching of cases and controls was addressed | 3 |
|  |  | (*e*) Describe any sensitivity analyses | 3 |
| Results | | | |
| Participants | 13* | (a) Report numbers of individuals at each stage of study—eg numbers potentially eligible, examined for eligibility, confirmed eligible, included in the study, completing follow-up, and analysed | 4 |
|  |  | (b) Give reasons for non-participation at each stage | N/A |
|  |  | (c) Consider use of a flow diagram | N/A |
| Descriptive data | 14* | (a) Give characteristics of study participants (eg demographic, clinical, social) and information on exposures and potential confounders | 4 |
|  |  | (b) Indicate number of participants with missing data for each variable of interest | 4 |
| Outcome data | 15* | Report numbers in each exposure category, or summary measures of exposure | 4 |
| Main results | 16 | (*a*) Give unadjusted estimates and, if applicable, confounder-adjusted estimates and their precision (eg, 95% confidence interval). Make clear which confounders were adjusted for and why they were included | 4 |
|  |  | (*b*) Report category boundaries when continuous variables were categorized | 4 |
|  |  | (*c*) If relevant, consider translating estimates of relative risk into absolute risk for a meaningful time period | NA |
| Other analyses | 17 | Report other analyses done—eg analyses of subgroups and interactions, and sensitivity analyses | 4-6 |
| **Discussion** | | | |
| Key results | 18 | Summarise key results with reference to study objectives | 6-10 |
| Limitations | 19 | Discuss limitations of the study, taking into account sources of potential bias or imprecision. Discuss both direction and magnitude of any potential bias | 10-11 |
| Interpretation | 20 | Give a cautious overall interpretation of results considering objectives, limitations, multiplicity of analyses, results from similar studies, and other relevant evidence | 11 |
| Generalisability | 21 | Discuss the generalisability (external validity) of the study results | NA |
| **Other information** | | | |
| Funding | 22 | Give the source of funding and the role of the funders for the present study and, if applicable, for the original study on which the present article is based | 12 |

**Table S2. Sensitivity analysis of clinical and laboratory parameters after exclusion of severity-defining variables.**

|  | **Univariate Model** | | **Multivariable Model** | |
| --- | --- | --- | --- | --- |
| **Variables** | **COR (95% CI)** | ***p*-value** | **AOR (95% CI)** | ***p*-value** |
| **WBCs (cells × 10^9^ /L)** | 0.80 (0.71-0.89) | <0.001 | 0.82 (0.67-1.01) | 0.051 |
| **Thrombocytes (cells × 10^9^ /L)** | 0.89 (0.86-0.91) | <0.001 | 0.89 (0.85-0.93) | <0.001 |
| **Hemoglobin (g/dL)** | 1.04 (0.97-1.12) | 0.261 |  |  |
| **Total bilirubin (μmol/L)** | 0.39 (0.32-0.47) | <0.001 | 0.39 (0.29-0.51) | <0.001 |
| **Albumin (g/L)** | 0.54 (0.47-0.61) | <0.001 | 0.55 (0.45-0.66) | <0.001 |
| **Globulin (g/L)** | 0.53 (0.46-0.60) | <0.001 | 0.59 (0.48-0.70) | <0.001 |
| **Pulse rate (BPM)** | 1.20 (1.11-1.30) | <0.001 | 1.10 (0.95-1.27) | 0.212 |

**Table S3. Sensitivity analysis of multivariable logistic regression excluding WHO 2009 warning-sign variables.**

| **Exposure variables** | **Univariate Model** | | **Multivariable**  **Model** | |
| --- | --- | --- | --- | --- |
|  | **COR (95% CI)** | **P-value** | **AOR (95% CI)** | **P-value** |
| **Socio-demographic** |  |  |  |  |
| **Marital Status** |  |  |  |  |
| Married | 2.04 (1.36-3.08) | <0.001 | 2.15 (1.37-3.39) | <0.001 |
| Unmarried | Reference |  | Reference |  |
| **Highest Education** |  |  |  |  |
| Postgraduate | 2.45 (0.80-7.51) | 0.110 | 2.49 (0.77-8.08) | 0.122 |
| College/University | 1.32 (0.74-2.34) | 0.340 | 1.41 (0.75-2.62) | 0.280 |
| High school or less | 1.33 (0.84-2.11) | 0.228 | 1.60 (0.96-2.66) | 0.071 |
| No education | Reference |  | Reference |  |
| **Monthly Income** |  |  |  |  |
| <15000 Taka | 1.10 (0.67-1.83) | 0.717 | 1.02 (0.60-1.76) | 0.942 |
| 15001-30000 Taka | 0.66 (0.36-1.20) | 0.172 | 0.68 (0.36-1.27) | 0.223 |
| 30000+ Taka | Reference |  | Reference |  |
| **Comorbidities** |  |  |  |  |
| **Obesity** |  |  |  |  |
| Yes | 1.05 (0.69-1.57) | 0.833 |  |  |
| No | Reference |  |  |  |
| **Diabetics** |  |  |  |  |
| Yes | 1.62 (0.70-3.65) | 0.248 |  |  |
| No | Reference |  |  |  |
| **Hypertension** |  |  |  |  |
| Yes | 1.41 (0.75-2.61) | 0.276 |  |  |
| No | Reference |  |  |  |
| **Chronic Pulmonary Disease** |  |  |  |  |
| Yes | 2.19 (1.18-4.07) | 0.013 | 2.16 (0.93-5.00) | 0.071 |
| No | Reference |  |  |  |
| **Ischemic Heart Disease** |  |  |  |  |
| Yes | 1.61 (0.77-3.34) | 0.197 | 0.85 (0.31-2.27) | 0.751 |
| No | Reference |  |  |  |
| **Signs and Symptoms** |  |  |  |  |
| **Diarrhea** |  |  |  |  |
| Yes | 1.11 (0.68-1.78) | 0.681 |  |  |
| No | Reference |  |  |  |
| **Headache** |  |  |  |  |
| Yes | 0.93 (0.59-1.51) | 0.780 |  |  |
| No | Reference |  |  |  |
| **Rash** |  |  |  |  |
| Yes | 1.08 (0.70-1.68) | 0.742 |  |  |
| No | Reference |  |  |  |
| **Chills and Rigors** |  |  |  |  |
| Yes | 0.98 (0.62-1.53) | 0.939 |  |  |
| No | Reference |  |  |  |
| **Nausea** |  |  |  |  |
| Yes | 2.06 (1.16-3.64) | 0.013 | 1.74 (0.93-3.22) | 0.078 |
| No | Reference |  |  |  |
| **Musculoskeletal Pain** |  |  |  |  |
| Yes | 1.20 (0.77-1.91) | 0.426 |  |  |
| No | Reference |  |  |  |
